# Editorial: Advancements in ultrasound for understanding the nervous system

**DOI:** 10.3389/fnhum.2024.1538535

**Published:** 2024-12-18

**Authors:** Vincent P. Clark

**Affiliations:** Psychology Clinical Neuroscience Center, Department of Psychology, The University of New Mexico, Albuquerque, NM, United States

**Keywords:** ultrasound, neuromodulation, neuroimaging, neurology, psychiatry, brain and behavior

As we approach the centennial of Harvey's pioneering description of ultrasound's effects on the nervous system (Harvey, 1929), this Research Topic explores cutting-edge transcranial ultrasound stimulation (TUS) research. It highlights advances that promise to transform our use of neuromodulation for cognitive augmentation and clinical therapeutics, and to increase our understanding of the human brain. TUS offers distinctive advantages over traditional neuromodulation techniques. Being mostly unconstrained by Maxwell's equations (Maxwell, 1873) and Lorentz force law (Feynman et al., 2006), TUS can achieve greater depth and focus, which is particularly valuable for targeting deeper brain networks underlying most psychiatric conditions. After accounting for skull and tissue lensing effects, TUS demonstrates superior spatial precision compared to other non-invasive neuromodulation methods, while also being safer and more cost-effective than more invasive methods of deep brain stimulation (Voges et al., 2007).

The breadth of clinical investigations is particularly promising. Matt et al. comprehensively reviewed TUS studies spanning a wide range of neuropsychiatric conditions, including pain management, dementia, movement disorders, epilepsy, schizophrenia, autism, and disorders of consciousness. Their analysis reveals an expanding therapeutic landscape, though they carefully note that while preliminary data are encouraging, larger, better controlled trials are needed, along with further research to better understand the mechanisms of TUS.

A particularly intriguing investigation by Peng et al. utilized functional MRI to explore TUS's effects on the brain's reward network. Their pilot clinical trial with healthy adults revealed fascinating neurological dynamics. The study demonstrated bilateral nucleus accumbens inhibition and increased functional connectivity between the nucleus accumbens and medial prefrontal cortex during transcranial focused ultrasound stimulation (tFUS). These findings hint at profound potential therapeutic implications, particularly for substance use disorders and related psychiatric conditions.

Technical innovations are critical to TUS's advancement, and Wilson, Riis, et al. describe significant strides in addressing a fundamental challenge: skull-induced ultrasound attenuation and defocusing. Their groundbreaking method directly measures and compensates for skull interference by transmitting and receiving ultrasound signals across the head. This approach enables more precise stimulation and opens new possibilities for targeted interventions. In a remarkable demonstration described in a second paper, Wilson, Parikh, et al. used this method to successfully deliver medication using perfluorocarbon nanodroplets, showing how precise ultrasound activation can mediate drug release in specific body regions. Interest in this new technique is reflected in its high score of all research outputs scored by Altmetric.

The exploration of this neuromodulation technique continues to expand in creativity and sophistication. Lybbert et al. investigated “Lstim,” an innovative approach combining magnetic fields with ultrasound to modulate neural activity. Their non-human primate study revealed transient neural inhibition at low pulse repetition frequencies (5 Hz), with no effects at higher frequencies. This research offers tantalizing insights into potential methods for targeted neural circuit manipulation, expanding our understanding of how combinations of non-invasive stimulation modalities can influence neural dynamics.

Lord et al. contributed another compelling piece of research applying tFUS to the posterior cingulate cortex (PCC), a key component of the default mode network. Their study involving 30 participants used comprehensive psychological assessments to explore neurological and cognitive effects of tFUS to the PCC. The results were striking: reduced functional connectivity in the default mode network, increased mindfulness measures, and intriguing alterations in participants' perceptions of ego and time. The study's significance is underscored by its placement in the top 5% of all research outputs scored on Altmetric, signaling a growing interest in mindfulness, and the potential importance of tFUS in understanding how changes in brain function and cognitive processes are related to mindfulness.

These investigations included here collectively illustrate the multifaceted potential of TUS. They also demonstrate a sophisticated balance between pure scientific discovery and practical technological development. Anticipated developments include creating protocols that are more efficacious and have longer-lasting effects, developing portable and more cost-effective TUS systems, and integrating TUS more comprehensively with neuroimaging technologies, included in some fashion in all of the experimental papers included in this Research Topic.

By combining neuroimaging with TUS, researchers are developing increasingly sophisticated protocols that offer unprecedented insights into brain function and potential therapeutic interventions. The further integration of TUS with neuroimaging may lead to many improvements, including a better understanding of brain function, especially the ability to confirm causal relationships suggested by neuroimaging studies, and identifying the mechanisms of TUS effects on brain and behavior. This may also lead to more effective personalized treatments, improving outcomes by using imaging to target neural fields in time and space for a specific individual, and using this to determine optimal protocols before treatment is started. Once treatment begins, further optimizing stimulation parameters based on neuroimaging data, and then later improving treatment efficacy in the long-term by monitoring treatment progress and refining stimulation parameters to maintain an optimal effect. The combination of imaging with TUS may also assist in identifying the mechanisms of any adverse effects, which could then be used to improve the overall safety of TUS. It should also be remembered that TUS can be used for both neuroimaging and neuromodulation. While its ability to image the brain is limited, further development may lead to better ultrasound based imaging and combined use for both targeting and stimulating specific brain areas.

Researchers are also exploring synergistic combinations with other treatment modalities, recognizing that the most effective interventions may emerge from interdisciplinary approaches. Critically, the success of these endeavors relies on continued collaboration. The most exciting and impactful progress occurs at the intersection of academic research, clinical practice, engineering innovation, and commercial development. By fostering these interdisciplinary partnerships, researchers can accelerate the translation of fascinating scientific discoveries into practical tools that address complex neurological and psychiatric challenges and to ultimately provide better treatments needed to reduce the suffering of patients.

The future of TUS for neuromodulation appears very promising. This Research Topic is not merely a collection of individual papers; it is a testament to human curiosity, technological innovation, and the relentless pursuit of knowledge. This will help us to gain a greater understanding of the brain—widely considered to be the most complex biological system known. As we stand on the threshold of transformative studies using TUS, these research efforts help to illuminate the path forward, offering hope for the future of cognitive augmentation and therapeutic treatments for disorders of brain and behavior.
